# Impact of adenotonsillectomy on sleep and behavioral outcomes in children: a longitudinal study

**DOI:** 10.1097/j.pbj.0000000000000309

**Published:** 2025-12-04

**Authors:** Francisco Alves de Sousa, Margarida Camanho, Marta Rios, Manuel Ferreira de Magalhães, Mariline Santos

**Affiliations:** a Otorhinolaryngology and Head & Neck surgery, Unidade Local de Saúde de Santo António, Porto, Portugal; b Doctoral Programme in Medical Sciences, Instituto de Ciências Biomédicas Abel Salazar, Porto, Portugal; c Instituto de Ciências Biomédicas Abel Salazar, Porto, Portugal; d Sleep Laboratory, Pediatrics Department of Centro Materno Infantil do Norte, Unidade Local de Saúde de Santo António, Porto, Portugal; e Pneumology Unit and Neonatology Unit, Pediatrics Department at Centro Materno Infantil do Norte (CMIN), Unidade Local de Saúde de Santo António, Porto, Portugal

**Keywords:** adenotonsillectomy, children, sleep-disordered breathing, obstructive sleep apnea, pediatric behavior, parasomnias, hyperactivity

## Abstract

**Background:**

Adenotonsillar hypertrophy is a prevalent pediatric condition that may affect children's health due to its association with obstructive sleep-disordered breathing (OSDB). This can negatively affect sleep quality, daytime behavior, neurocognitive development, and growth. This study aimed to evaluate the impact of adenotonsillectomy on children's sleep habits and behavior. A secondary objective was to explore differences in outcomes between children undergoing surgery for recurrent upper respiratory tract infections (URTIs) versus those with OSDB.

**Methods:**

A prospective longitudinal observational study was conducted from December 2023 to December 2024, enrolling 28 children aged 2–10 years undergoing adenotonsillectomy. Sleep and behavioral changes were assessed preoperatively and postoperatively (3–6 months) using the validated Children's Sleep Habits Questionnaire—adapted Portuguese version and the Strengths and Difficulties Questionnaire—Portuguese version. Paired samples *t*-tests and general linear models were used for statistical analysis.

**Results:**

Significant improvements were observed in several sleep parameters: sleep duration, parasomnias, sleep-related breathing disorders, daytime sleepiness, and the total sleep disturbance index (*P* < 0.001 for all). In the behavioral domain, hyperactivity was the only subscale to show statistically significant improvement (*P* = 0.028). Surgical indication (OSDB vs. recurrent URTIs) did not significantly influence most outcomes, except for sleep-related breathing disorders, where greater improvement was noted in the OSDB group, as expected.

**Conclusion:**

Adenotonsillectomy positively affects multiple dimensions of sleep, particularly reducing parasomnia episodes and hyperactivity symptoms, regardless of surgical indication. These findings underscore the importance of early recognition and management of pediatric adenotonsillar disorders to promote healthy sleep, improved daytime functioning, and long-term psychosocial development.

## Abstract 2

Supplemental Digital Content is Available in the Text.

## Introduction

Adenotonsillar hypertrophy (ATH) is a highly prevalent condition in the pediatric population and constitutes a major concern in pediatric otolaryngology. The lymphoid tissues forming Waldeyer's ring, particularly the palatine and pharyngeal tonsils, are critical for immune defense in the upper respiratory tract.^1,2^ However, in some children, abnormal growth of these structures can lead to upper airway obstruction and significant complications affecting various physiological functions, especially during sleep.^3,4^

One of the most critical consequences of this hypertrophy is its strong association with obstructive sleep-disordered breathing (OSDB).^2,5^ Pediatric OSDB is a condition that negatively affects sleep quality, daytime behavior, neurocognitive development, and normal child growth.^3,5-7^ The mechanical obstruction caused by enlarged adenoids and tonsils results in fragmented sleep and intermittent hypoxemia, which are believed to be responsible for a range of behavioral and cognitive impairments observed in affected children.^8-10^ For instance, children with OSDB often present with symptoms such as hyperactivity, inattention, and learning difficulties, leading to poor school performance.^8,11,12^ Furthermore, OSDB is increasingly recognized as a contributing factor to parasomnias, including night terrors and sleepwalking, due to frequent nocturnal arousals and disrupted sleep architecture.^8,12-14^

Adenotonsillectomy (AdT) is the most commonly performed surgical procedure in children younger than 15 years.^15^ It represents the first-line treatment for pediatric OSDB in otherwise healthy children with ATH.^4,16^ Beyond OSDB, these procedures' indications also include recurrent or chronic upper respiratory tract infections (URTIs).^4,16^ Growing evidence highlights the significant and lasting benefits of AdT, encompassing improvements in behavior, neurocognitive performance, school outcomes, and overall quality of life.^17^ AdT has also been linked to reduced incidence of upper respiratory infections, lower healthcare utilization, and decreased school absenteeism.^4,5,18,19^

Despite the recognized benefits, understanding of how AdT specifically affects sleep and behavioral patterns across different primary indications (obstructive vs. infectious) in pediatric populations is still evolving. This study aims to prospectively investigate the evolution of children's sleeping habits and behavioral patterns, as reported by their caregivers, at baseline and 3–6 months following AdT performed due to recurrent URTIs, clinical suspicion of OSDB, or both. By assessing these outcomes before and after surgery, this research seeks to provide deeper insight into the benefits of this common procedure, ultimately contributing to better-informed clinical decision-making and improved quality of life for pediatric patients.

## Methods

### Study design and participants

This was a prospective, longitudinal, and observational study conducted at Unidade Local de Saúde de Santo António, Porto, Portugal. Children aged 2–10 years who underwent outpatient AdT between December 2023 and May 2024 were included. The study was approved by the local Ethics Committee—approval 2023-321 (248-CAC/293-CE) and 2021.234(186-DEFI/194-CE)—and informed consent was obtained from all caregivers.

### Inclusion criteria

Patients aged 2–10 years scheduled for primary outpatient otolaryngology (ORL) surgery involving total AdT with surgical indication based on clinical suspicion of OSDB and/or recurrent URTIs.

### Exclusion criteria

Patients with prior ORL procedures (including previous tonsillectomy/adenoidectomy), surgical indications unrelated to recurrent URTIs or suspected OSDB, incomplete clinical information or any comorbidity contraindicating outpatient procedure, namely, congenital craniofacial anomalies, cardiovascular or pulmonary disease (including asthma), obesity, neuromuscular diseases, known genetic syndromes (e.g., Down syndrome). Finally, all drop-outs were excluded from the final sample.

### Data collection

Demographic, clinical, and surgical data, including child's sex, date of surgery, and surgical indication (recurrent URTIs, clinical features of OSDB, or both), were extracted from the electronic health records. Data were pseudonymized for confidentiality.

### Assessment instruments

Caregivers completed two validated questionnaires at baseline (presurgery) and 3–6 months postoperatively:

- Children's Sleep Habits Questionnaire—abbreviated Portuguese version (CSHQ-PT)^20^ (Appendix A, http://links.lww.com/PBJ/A46): This 33-item questionnaire assesses various sleep behaviors over the past week, grouped into eight subscales: Bedtime Resistance, Sleep Onset Delay, Sleep Duration, Sleep Anxiety, Night Wakings, Parasomnias, Sleep-Disordered Breathing, and Daytime Sleepiness. A total sleep disturbance score is derived, with higher scores indicating greater severity.
- Strengths and Difficulties Questionnaire—Parent´s Portuguese version (SDQ-Por)^21^ (Appendix B, http://links.lww.com/PBJ/A47): This 25-item questionnaire evaluates five subscales: Emotional Symptoms, Conduct Problems, Hyperactivity or Inattention, Peer Relationship Problems, and Prosocial Behavior. A total difficulties score is calculated from the first four subscales, with higher scores reflecting more severe difficulties.

### Surgical technique

AdT was performed under general anesthesia, with adenoidectomy accomplished through curettage followed by compression and total extracapsular tonsillectomy conducted using cold steel dissection and absorbable hemostatic sutures.

### Statistical analysis

Data were analyzed using IBM SPSS Statistics version 29.0. Paired-samples *t*-tests were used to compare preoperative and postoperative scores for each subscale and total scores. General linear models with a within-subject factor (time) and a between-subject factor (surgical indication) were used to explore interactions. A significance threshold of *P* < 0.05 was used. Cohen's d effect sizes were calculated for the magnitude of change.

## Results

### Participant flow and characteristics

#### Sample selection

**Figure 1.**
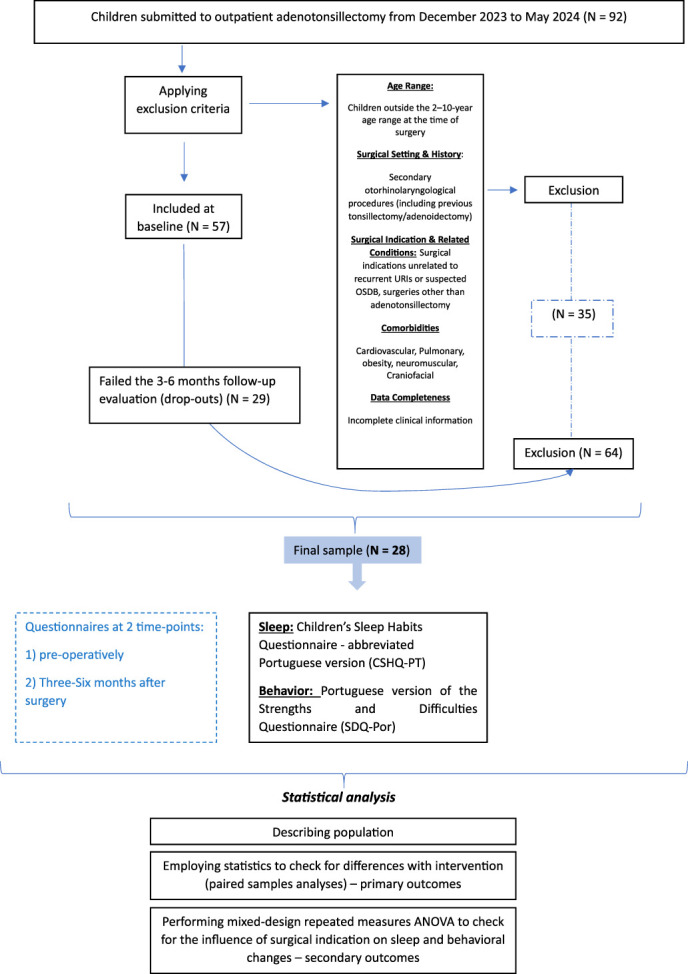
Sample selection and statistical analysis design.

Of 92 patients initially screened for the study, 35 were excluded due to meeting the exclusion criteria, and an additional 29 were lost to follow-up, yielding a final cohort of 28 participants with both preoperative and postoperative data (Fig. 1).

#### Follow-up period

Within the follow-up period (3–6 months postoperatively), the mean response time of the caregivers to the questionnaires was about 4 months after surgery (4.41 ± 1.52 months).

#### Age and sex

The mean age of participants at surgery was 5.36 ± 3.25 years, with 17 males (60.7%) and 11 females (39.3%). No statistically significant difference in age at the time of surgery between males and females was noted (males: 5.35 ± 2.42 vs. females: 5.36 ± 4.36 years, *P* = .994).

#### Surgical indication

All patients underwent total AdT. Most patients (n = 20, 71.4%) were indicated for surgery due to clinical features of OSDB. Recurrent URTIs indicated surgery in 8 children (28.6%).

#### Sleep schedule

Preoperatively, the mean bedtime during the week was 21:24 (±34 minutes), shifting later to 22:07 (±39 minutes) on weekends. The mean awakening time before surgery was 07:30 (±46 minutes) on weekdays and 08:41 (±68 minutes) on weekends. The average duration of during-night awakenings before the surgical intervention was 10 minutes and 12 seconds (±2 minutes).

### Exploring the primary outcome: the impact of surgery on sleep and behavior

#### Sleep

Scores were obtained from the responses to Children's Sleep Habits Questionnaire. Table 1 presents the main results. Statistically significant improvements (*P* < .05) were observed postoperatively in several sleep parameters. Specifically, there was a significant reduction in the scores for Sleep Duration (meaning less fragmentation and longer periods of sleep after surgery), Parasomnias, Sleep-Related Breathing Disorder, Daytime Sleepiness, and the Total Sleep Disturbance Index.

**Table 1:** Comparative analysis of preoperative and postoperative values of sleep and behavior

	Subscale score*	Mean/Median (SD, P25–P75)		
		Preoperative	Postoperative	*P* †
SLEEP (Children's Sleep Habits Questionnaire—abbreviated Portuguese version [CSHQ-PT])	Resistance to going to bed‡	10.4 ± 3.1	9.4 ± 2.7	0.066
	Sleep onset§	1.6 ± 0.8	1.4 ± 0.7	0.177
	Sleep duration‖	5.1 ± 2	3.3 ± 0.7	**<0.001**
	Sleep-related anxiety¶	7.7 ± 2.4	7.1 ± 2.5	0.132
	Night wakings#	5.3 ± 1.5	4.8 ± 1.2	0.116
	Parasomnias**	10.8 ± 2.9	8.3 ± 3.1	**<0.001**
	Sleep-related breathing disorder††	7.4 ± 2.1	3.2 ± 0.5	**<0.001**
	Daytime sleepiness‡‡	14.9 ± 3	13.2 ± 3.1	**0.003**
	Total score (sleep disturbance index)§§	58.7 ± 9.8	46.9 ± 6.5	**<0.001**
BEHAVIOR (Portuguese version of the Strengths and Difficulties Questionnaire [SDQ-Por])	Problems			
	Emotional symptoms	3 ± 2.5	2.6 ± 2.4	0.405
	Behavioral problems	1.9 ± 2	1.7 ± 1.5	0.578
	Hyperactivity	5 ± 2.7	4 ± 2.6	**0.028**
	Peer relationship problems	1.2 ± 1.8	1 ± 1.6	0.246
	Total difficulties score‖‖	11.1 ± 6.6	9.2 ± 6	0.406
	Prosocial behavior	8.7 ± 1.8	8.9 ± 1.7	0.078

* Calculated as the sum of the scores of the items belonging to that specific subscale.

† *P*-values describe the analysis by using paired samples *t* test, bold for statistically significant changes.

‡ SLEEP: Sum of items 1, 3, 4, 5, 6, and 8.

§ SLEEP: Item 2.

‖ SLEEP: Sum of items 9, 10, and 11.

¶ SLEEP: Sum of items 5, 7, 8, and 21.

# SLEEP: Sum of items 16, 24, and 25.

** SLEEP: Sum of the items 12, 13, 14, 15, 17, 22, and 23.

†† SLEEP: Sum of items 18, 19, and 20.

‡‡ SLEEP: Sum of items 26, 27, 28, 29, 30, 31, 32, and 33.

§§ SLEEP: Sum of the scores of the 33 items— a higher value corresponds to more problems. Note: this index is not equivalent to the sum of the subscale scores, as 2 items appear in 2 subscales (items 5 and 8).

‖‖ BEHAVIOR: Each score results from the sum of items in each scale (5 items per scale). Total Difficulties Score—obtained by summing the total scores of all the scales with the exception of the prosocial scale. In this way, the resulting score can range between 0 and 40.

Conversely, no statistically significant changes were found in Resistance to Going to Bed, Sleep Onset, Sleep-Related Anxiety, and Night Wakings after the surgical intervention.

#### Behavior

Regarding behavior, as assessed by the Portuguese version of the Strengths and Difficulties Questionnaire (SDQ-Por), only hyperactivity showed a statistically significant reduction postoperatively. No significant changes were observed in Emotional Symptoms, Behavioral Problems, Peer Relationship Problems, Total Difficulties Score, or Prosocial Behavior after surgery (Table 1).

### Exploring the secondary outcome: influence of surgical indication on sleep and behavior changes

**Figure 2.**
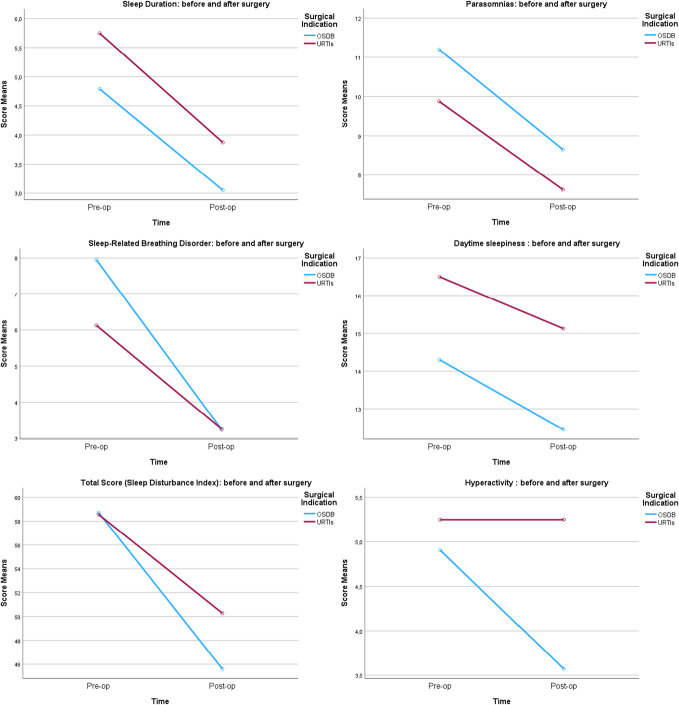
Significant preoperative and postoperative changes in sleep and behavior (by surgical indication).

After the identification of significant preoperative to postoperative changes in several sleep and behavior parameters (Sleep Duration, Parasomnias, Sleep-Related Breathing Disorder, Daytime Sleepiness, Total Sleep Disturbance Index, and Hyperactivity), a more detailed analysis was conducted to explore the impact of surgical indication (OSDB vs. URTIs) on these outcomes. A mixed-design repeated measures ANOVA was used, with surgical indication as the between-subjects factor and the preoperative and postoperative measurements as the within-subjects factor (Fig. 2). For the majority of sleep and behavioral outcomes examined in the multivariate analysis, surgical indication (OSDB vs. URTIs) did not significantly influence the extent of preoperative to postoperative changes. However, as expected, a notable contrast was observed for the Sleep-Related Breathing Disorder score: Patients with OSDB experienced significantly greater improvements in this domain following the intervention compared with those with URTIs. The detailed results for all parameters are presented in the following subsections.

#### Sleep duration

Concerning sleep duration (SD), there was a significant main effect of AdT on SD values, F (1,25) = 19.01, *P* < 0.001. SD score was significantly lower after (M = 3.464, SE = 0.414, 95% CI [3.221, 3.207]) than before surgery (M = 5.27, SE = 0.118, 95% CI [4.416, 6.123]).

Nevertheless, the main effect of surgical indication was not significant (F [1,25] = 3.98, *P* = 0.057), and no interaction was observed between surgery effects on outcome and surgical indication F (1,25) = 0.028, *P* = 0.869.

#### Parasomnias

Regarding Parasomnias, there was a significant main effect of AdT on Parasomnias score, F (1,26) = 20.083, *P* < 0.001. Parasomnias score was significantly lower after (M = 8.137, SE = 0.431, 95% CI [7.251, 9.024]) than before surgery (M = 10.537, SE = 0.597, 95% CI [9.310, 11.765]).

Nevertheless, the main effect of surgical indication was not significant (F [1,26] = 1.729, *P* = 0.2) and no interaction was observed between surgery effects on outcome and surgical indication F (1,26) = 0.078, *P* = 0.782.

#### Sleep-related breathing disorder

There was a significant main effect of AdT on sleep-related breathing disorders (SRBD) score, F (1,25) = 70.707, *P* < 0.001. SRBD score was significantly lower after (M = 3.243, SE = 0.109, 95% CI [3.019, 3.468]) than before surgery (M = 7.036, SE = 0.597, 95% CI [6.160, 7.912]).

The main effect of surgical indication was significant (F [1,25] = 4.495, *P* = 0.044), and a nearly significant interaction was observed between surgery effects on SRBD and surgical indication F (1,25) = 4.140, *P* = 0.053.

#### Daytime sleepiness

Regarding daytime sleepiness (DS), AdT had a significant main effect on DS score, F (1,26) = 7.729, *P* = 0.010. DS score was significantly lower after (M = 13.788, SE = 0.606, 95% CI [12.542, 15.033]) than before surgery (M = 15.400, SE = 0.609, 95% CI [14.149, 16.651]).

The main effect of surgical indication was significant (F [1,26] = 5.215, *P* = 0.031), but no significant interaction was observed between surgery effects on DS and surgical indication F (1,26) = 0.168, *P* = 0.686.

#### Total sleep disturbance index

Concerning Total Sleep Disturbance Index (TSDI), there was a significant main effect of AdT on TSDI score, F (1,23) = 33.196, *P* < 0.001. TSDI score was significantly lower after (M = 47.948, SE = 1.397, 95% CI [45.058, 50.839]) than before surgery (M = 58.647, SE = 2.231, 95% CI [54.032, 63.261]).

The main effect of surgical indication was not significant (F [1,23] = 0.492, *P* = 0.490), and no significant interaction was observed between surgery effects on TSDI and surgical indication F (1,26) = 0.168, *P* = 0.686.

#### Hyperactivity

Concerning Hyperactivity, no significant main effect of AdT was observed on Hyperactivity score, F (1,27) = 2.144, *P* = 0.155. Hyperactivity score was lower after (M = 4.411, SE = 0.527, 95% CI [3.330, 5.492]) than before surgery (M = 5.077, SE = 0.547, 95% CI [3.955, 6.200]).

The main effect of surgical indication was not significant (F [1,27] = 1.082, *P* = 0.308), and no significant interaction was observed between surgery effects on Hyperactivity and surgical indication F (1,27) = 2.144, *P* = 0.155.

## Discussion

This study demonstrates that AdT significantly improves multiple dimensions of sleep and reduces hyperactivity symptoms in children aged 2–10 years. These findings are particularly relevant for pediatric otolaryngology, as they highlight the broader benefits of these common procedures beyond merely alleviating physical airway obstruction and/or URTIs.

The most striking improvements were observed in sleep-related breathing disorder ‘symptoms, parasomnias episodes, sleep duration, and daytime sleepiness. This is consistent with existing literature demonstrating the effectiveness of AdT in resolving upper airway obstruction, which is a primary cause of sleep fragmentation in children with OSDB.^22,23^ The observed reduction in sleep-related breathing disorders' symptoms reflects the direct impact of surgery on the anatomical source of obstruction.^24^ Improvements in sleep duration and daytime alertness further suggest that surgery promotes more continuous and restorative sleep, with positive implications for daily functioning.

A key finding was the significant reduction in parasomnia symptoms. Parasomnias, such as night terrors and sleepwalking, are often exacerbated by sleep fragmentation and micro-arousals linked to obstructive respiratory events.^8,14^ By eliminating the anatomical obstruction, AdT appears to stabilize deep sleep cycles and reduce autonomic nervous system activation, thereby explaining the observed improvement in parasomnia scores. This is consistent with previous research showing a strong correlation between OSDB and parasomnias, and their resolution after surgery.^7,8,13,25^ Importantly, this improvement in parasomnias occurred regardless of the surgical indication (OSDB vs. URTIs), suggesting a broad positive effect of the intervention on sleep stability. This has significant clinical implications, as parasomnias can be highly disruptive for both children and families.^26^ The overall improvement in the Total Sleep Disturbance Index reinforces the global enhancement of sleep quality postsurgery.

While significant improvements were seen in many sleep parameters, some subscales (e.g., bedtime resistance, sleep onset) did not reach statistical significance. This may be attributed to the multifactorial nature of these parameters, which can be influenced by behavioral, emotional, and environmental factors not directly targeted by surgery. In such cases, surgical intervention might need to be complemented by targeted behavioral strategies.

In the behavioral domain, only hyperactivity showed a statistically significant reduction. This is particularly relevant given the phenotypic overlap between OSDB and Attention Deficit Hyperactivity Disorder (ADHD), where poor sleep quality, hypoxemia, and nocturnal microarousals can exacerbate hyperactivity and attention deficits.^8,9^ This fact can be explained by altered neurotransmitter regulation in the insular cortex, with lower levels of Gamma-Aminobutyric Acid (GABA), an inhibitory neurotransmitter, and higher levels of glutamate, an excitatory neurotransmitter. Brain structural changes may also ensue, since intermittent hypoxia and hypercapnia can contribute to brain oxidative stress, with consequent neuronal injury and vascular inflammation, leading to reduced cortical thickness, particularly in the prefrontal cortex and hippocampus, critical areas in the regulation of behavior, executive function and memory.^8,9^ The restoration of healthy sleep patterns postsurgery likely contributes to improved neurobehavioral regulation, leading to a reduction in hyperactive symptoms. This suggests that AdT can act as an indirect modulator of behavior, especially when underlying OSDB is present. The lack of significant changes in other behavioral subscales may reflect the complex multifactorial nature of child behavior, influenced by various familial, environmental, and individual factors. In addition, the relatively short follow-up period may have limited the detection of more sustained behavioral changes.

Interestingly, the surgical indication (OSDB vs. recurrent URTIs) did not significantly influence the degree of improvement for most variables. This suggests that AdT can confer sleep benefits even in children whose primary indication is not directly related to nocturnal respiratory symptoms. URTIs can significantly affect both the quantity and quality of sleep.^27,28^ This may help to explain the similar outcomes in the two patient groups. In addition, it is common for patients to experience both OSDB and URTIs, as these conditions can be interconnected. This relationship suggests a broader underlying inflammatory state or cycle, where one condition may exacerbate the other.^27,28^ The overall findings, suggesting therapeutic efficacy across different clinical subgroups, support a symptom-oriented approach to surgical decision-making in children with disrupted sleep.

### Limitations

This study has limitations, including a modest sample size (N = 28), which may limit statistical power and generalizability. A high dropout rate (32%) could introduce selection bias. The absence of a control group restricts causal inferences, and reliance on parental self-report introduces potential perception bias. The lack of objective sleep measures, such as polysomnography, limits the physiological characterization of observed phenomena. Finally, the 3–6-month follow-up period may have been too short to detect long-term emotional or social functioning changes.

## Conclusion

This study reinforces the multidimensional benefits of tonsillectomy and adenoidectomy in the pediatric population, particularly for children with obstructive sleep-disordered breathing and recurrent upper respiratory tract infections. Surgical intervention significantly improved various sleep parameters, with a notable reduction in parasomnias. This may translate the procedure's effectiveness in restoring deeper and more stable sleep cycles, enhancing overall sleep architecture and quality of life. Furthermore, the improvement in hyperactivity symptoms suggests a link between restored sleep quality and improved neurobehavioral regulation, underscoring sleep's crucial role in children's executive functioning. The efficacy of the procedure was often evident across surgical indications (obstructive vs. infectious), reinforcing a symptom-oriented approach to surgical decision-making. Early recognition and management of OSDB in children through AdT are crucial, as this can offer benefits extending well beyond respiratory improvement.
